# Random forest algorithms to classify frailty and falling history in seniors using plantar pressure measurement insoles: a large-scale feasibility study

**DOI:** 10.1186/s12877-022-03425-5

**Published:** 2022-09-12

**Authors:** Emi Anzai, Dian Ren, Leo Cazenille, Nathanael Aubert-Kato, Julien Tripette, Yuji Ohta

**Affiliations:** 1 Faculty of Engineering, Nara Women’s University, Nara, Japan; 2 Department of Cooperative Major in Human Centered Engineering, Graduate School of Humanities and Sciences, Ochanomizu University, Tokyo, Japan; 3 Department of Information Sciences, Ochanomizu University, Tokyo, Japan; 4 Comprehensive Research Building, 6th floor, room 613, Bunkyo, Tokyo 112-8610 Japan; 5 Department of Human-Environmental Science, Faculty of Human Life and Environmental Sciences, Ochanomizu University, Tokyo, Japan; 6 Faculty of Core Research Natural Science Division, Ochanomizu University, Tokyo, Japan

**Keywords:** Frailty, Fall risk, Aging, Plantar pressure, Smart-insole, Balance, Walking, Gait analysis, Random forest classifier

## Abstract

**Background:**

Frailty and falls are two adverse characteristics of aging that impair the quality of life of senior people and increase the burden on the healthcare system. Various methods exist to evaluate frailty, but none of them are considered the gold standard. Technological methods have also been proposed to assess the risk of falling in seniors. This study aims to propose an objective method for complementing existing methods used to identify the frail state and risk of falling in older adults.

**Method:**

A total of 712 subjects (age: 71.3 ± 8.2 years, including 505 women and 207 men) were recruited from two Japanese cities. Two hundred and three people were classified as frail according to the Kihon Checklist. One hundred and forty-two people presented with a history of falling during the previous 12 months. The subjects performed a 45 s standing balance test and a 20 m round walking trial. The plantar pressure data were collected using a 7-sensor insole. One hundred and eighty-four data features were extracted. Automatic learning random forest algorithms were used to build the frailty and faller classifiers. The discrimination capabilities of the features in the classification models were explored.

**Results:**

The overall balanced accuracy for the recognition of frail subjects was 0.75 ± 0.04 (F1-score: 0.77 ± 0.03). One sub-analysis using data collected for men aged > 65 years only revealed accuracies as high as 0.78 ± 0.07 (F1-score: 0.79 ± 0.05). The overall balanced accuracy for classifying subjects with a recent history of falling was 0.57 ± 0.05 (F1-score: 0.62 ± 0.04). The classification of subjects relative to their frailty state primarily relied on features extracted from the plantar pressure series collected during the walking test.

**Conclusion:**

In the future, plantar pressures measured with smart insoles inserted in the shoes of senior people may be used to evaluate aspects of frailty related to the physical dimension (e.g., gait and balance alterations), thus allowing assisting clinicians in the early identification of frail individuals.

**Supplementary Information:**

The online version contains supplementary material available at 10.1186/s12877-022-03425-5.

## Background

Frailty and falls are two adverse characteristics of aging that negatively impact the well-being of senior people. Frailty results from cumulative declines across multiple physiological systems, causing vulnerability to adverse outcomes and increasing the risk of dependency in older adults [1]. The global prevalence of frailty in people aged ≥50 years is estimated to be between 12 and 24%, depending on the diagnostic criteria [2]. Falls are a significant health issue in older people that can result from a combination of intrinsic and extrinsic factors. Physical weakness due to frailty is one of these factors [3]. Indeed, Cheng and Chang (2017) have reported a higher risk of falls in frail people compared to their robust counterparts (odd ratio: 2.50; 95% confidence interval: 1.58–3.96) in a sample of 102,130 individuals aged over 65 years [3]. Falls lead to injuries ranging from simple bruises to more severe fractures and significantly burden healthcare systems. In the current context of aging societies, public health strategies to promote healthy aging need to be prioritized [4]. These strategies may include early diagnosis of frailty and identifying falling risks to develop interventions to slow down the frailty process and prevent falls [5, 6].

### Frailty diagnosis

During the past two decades, the development of robust diagnostic tools for the early detection of frailty in aging individuals has been the object of many efforts by the geriatrician community [7]. Several methods have been developed to evaluate frailty in all dimensions (i.e., physiological, physical, cognitive, and social). In Japan, the Kihon Checklist is the primary choice for evaluating frailty [8]. This questionnaire consists of a self-reporting survey of 25 yes/no questions that cover 7 dimensions of frailty, i.e., instrumental and social activities of daily living, physical functions, nutritional status, oral function, cognitive function, and depressive mood (Supplementary Material 1). To complement the existing methods, researchers have been interested in using technological tools to analyze postural balance and gait stability and find early markers of frailty [7, 9–15]. Greene et al. [15] achieved 84 and 94% accuracy in classifying pre-frail and healthy women and men, respectively. In this study, 124 subjects performed the time up and go (TUG), sit-to-stand, and standing balance tests, with accelerometer sensors attached to different body parts for each test (TUG: 2 sensors on each shin, sit-to-stand: thigh, standing balance: waist). Despite promising results, previous studies come with several limitations [7]. First, they have usually been conducted on a limited number of subjects. Second, the proposed evaluation systems may involve a network of sensors attached to different parts of the body rather than one single easy-to-use device. Third, these systems are essentially based on inertial sensors and only Chkeir et al. (2016) have explored ground reaction sensing technologies [12]. Fourth, only a few of them integrate statistical learning-based algorithms [7, 13, 15]. Finally, technological tools are often used in conjunction with functional tests to produce a more robust testing environment [13–15]. To summarize, easy-to-use technology able to perform frailty assessments in free-living conditions or short walking segments are yet to be developed.

### Fall risk assessment

Several approaches have also been considered for assessing the risk of falling in older adults. First, functional tests, such as the TUG, have been diverted from their initial use to predict future falling events in senior people [16, 17]. The reports showed contrasting results. Second, the evaluation of gait stability by measuring a panel of spatial-temporal parameters (cadence, stride length, speed, etc.) has been suggested. Early observations are promising. Hausdorff et al. (2001) identified gait stride time variability as a good predictor of future falling events in older adults living in community dwellings [18]. However, the limited number of studies associated with a wide variability of methodologies and results does not allow the identification of standardized tests [19]. Finally, several studies have analyzed plantar pressures during walking trials [20–23]. Svoboda et al. (2017) used a prospective trial protocol. They observed a statistical relationship between the inter-step variability of displacement of the center of pressure (COP) in the medial-lateral direction and future falling events in senior people [22]. A greater variability was found among fallers compared to non-fallers. Despite these promising observations, space and cost are two significant challenges in implementing multiple-step walking trials in hospitals or community dwellings. The installation of walkways featuring force plates to collect spatiotemporal gait parameters requires a large space. Alternatively, portable devices such as the F-scan insole (Tekscan Inc., MA, USA) can be used to measure plantar pressure. In practice, the high cost associated with the inclusion of hundreds of pressure-sensitive transducers on each insole has limited its use to research trials only. The recent development of non-expensive wearable plantar pressure measurement insoles might help increase the portability and accessibility of COP measurement methods while providing access to a wide range of gait-sensitive parameters [24–26].

The present study addresses the feasibility of using a simple 7-sensor plantar measurement insole inserted in Velcro shoes [26–28] to predict frailty and evaluate the risk of falling in older adults. Plantar pressure data were collected from 774 senior Japanese people during a standing balance test and a 20 m walking trial. We hypothesized that statistical learning models trained with spatial-temporal and COP parameters extracted from the plantar pressure data collected during these two tests allow classifying subjects relative to their frailty status and history of falling events. A secondary analysis aims to elucidate the features of plantar pressure that may play the most crucial role in the classification.

## Method

### Plantar pressure measurement insole

**Fig. 1:**
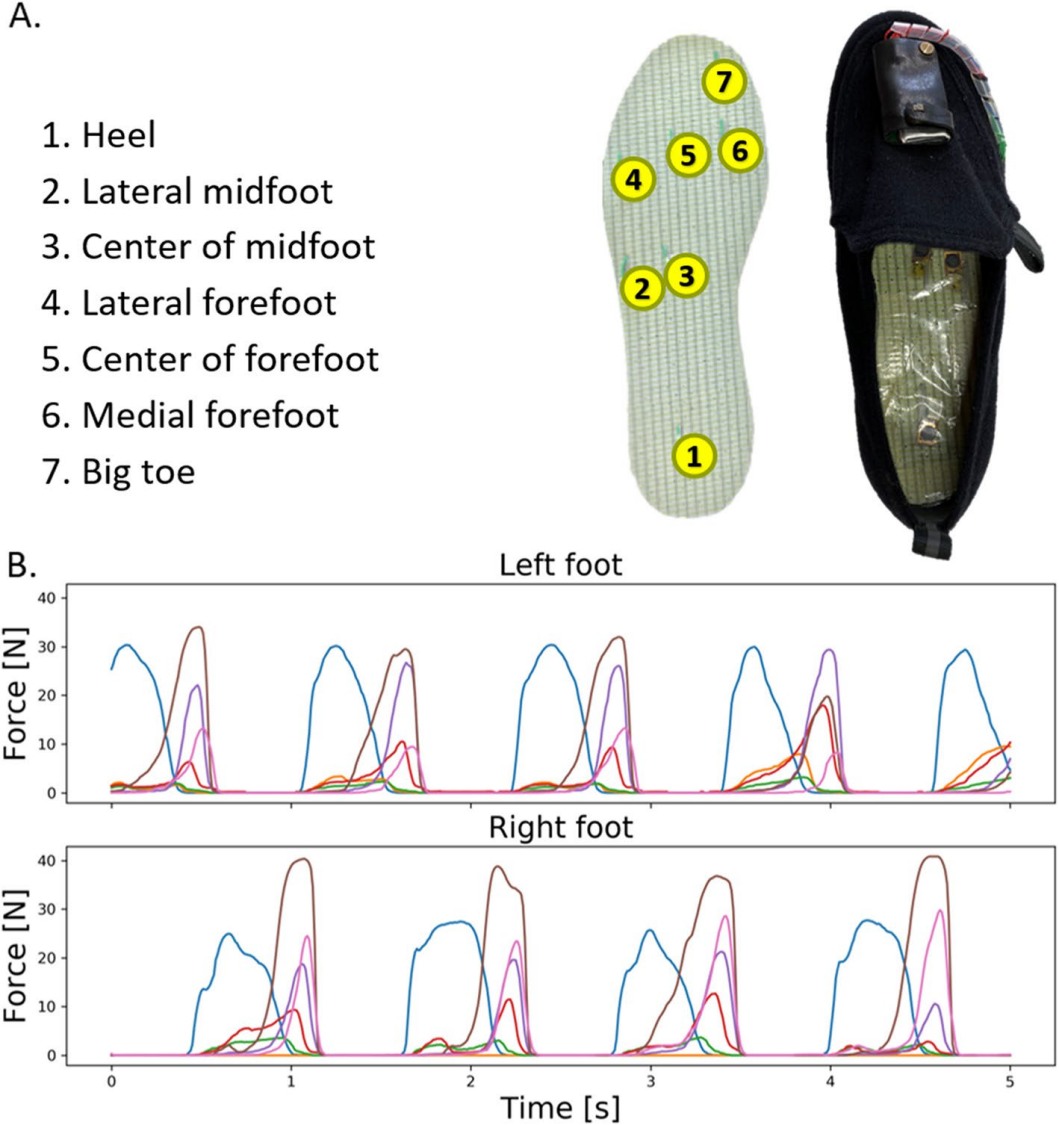
Overview of the 7-sensor plantar pressure measurement insole device and output. **A**: Seven pressure sensors are inserted in a 2 mm-thick hygienic shoe insole. The insole is inserted in a commercial Velcro shoe, and the data acquisition unit is attached using an additional piece of Velcro on the tong. **B**: A five-second example of plantar pressure data was collected from a walking test

This study measured plantar pressure with a 7-sensor plantar pressure measurement insole device developed at Ochanomizu University and described elsewhere [26–29] The insole consists of a 2 mm thick shoe insole with seven pressure-sensitive conductive rubber sensors. The sensors respond to force stimuli ranging from 25 to 550 kPa, and the output ranges from 0 to 3.0 V. The analog-to-digital converter uses a 10-bit scale. Data from mechanical load repeatability tests are available in Supplementary Material 2. The data were sampled at 100 Hz. The sensors are located in the heel, lateral midfoot, center of the midfoot, lateral forefoot, center of the forefoot, medial forefoot, and big toe (Fig. 1). The 7-sensor insole has been reported to provide valid COP measurements [28]. A wireless data transmission unit (Bluetooth Ver. 2.0, Class 1) was connected to each insole. The system is reusable and fully portable, allowing real-time recording during normal ambulatory activities. The insoles (left and right feet) were inserted into commercial Velcro shoes (Kaihoshugi M003, Asahi Corp., Japan). The shoes were lightweight and easy to use. They have a stiff midsole with a 2 mm medial drop in the heel area and an additional 1 mm drop in the medial forefoot region. They are also commonly used in community dwellings. The insoles were available from 22 cm to 28 cm to fit each participant’s foot size. This material is shown in Fig. 1. For one given shoe size, the same pair of insoles was used for the whole experiment. The sensor calibration was verified every 4 months using standardized mechanical loads. No significant deviation was noted over time, emphasizing the durability of the material and ensuring measurement consistency over the whole experimental period.

Apart from the 7-sensor plantar pressure measurement insole devices, no other sensing technology was used in the present study.

### Subject recruitment and data collection

A total of 774 senior people who could walk independently and live in both rural and urban areas of Japan participated in the study. Exclusion criteria at the time of recruitment included (i) presence of paresis or any other musculoskeletal diseases, (ii) history of heart disease or stroke, and (iii) history of any surgery in the 6 months preceding the measurement. Some subjects were recruited in Shiki city, Saitama prefecture, Japan, with the help of the municipality health and welfare administration. Other subjects were recruited in the northwestern part of Akita Prefecture through Peaberry Corporation (Ogata city, Japan), a local healthcare provider. Measurements were performed at the Shiki City Health Promotion Center, the Shiki City General Welfare Center, the Shiki City Fourth Elementary School, and in the premises of the Peaberry Corporation. Measurements were performed between 2014 and 2016.

The experimental visits were conducted as follows. First, anthropometric data were collected for all participants. Second, the subjects put on a pair of the above-described Velcro shoes equipped with two 7-sensor insoles and performed a 45 s standing balance test and a 20 m walking trail test. In the standing test, subjects kept their feet in a specified position with a heel distance of 80 mm and tips of shoe toe distance of 120 mm. They were asked to gaze at one fixed circle of 10 cm diameter attached at eye height on a wall located 1.5 m before them [30]. The basic instruction consisted in asking the subjects to maintain the posture for “approximatively 1 minute”. The last 45 seconds of the test were used for the analysis. In the walking experiment, the subjects walked straight for 10 m at a self-selected pace toward a goal materialized by a mark on the ground. Then, the subjects were asked to turn around and come back to the starting point. On their way back to the starting point, again, the subjects walked straight for 10 m at a self-selected pace. Data were collected and analyzed for the two 10-m walking segments. Data were collected at a sampling rate of 100 Hz.

During the interviews, 147 subjects indicated that they had experienced at least one falling event within the 12 months preceding the visit. The subjects confirmed the results from the Japanese frailty Kihon checklist obtained from Shiki City Health and Welfare Administration and Peabearry Corporation. A total of 203 subjects were considered frail. Frailty was assessed using the 20 first questions of the Kihon checklist and the original “4-criteria treatment method” described in Supplementary Material 1 and elsewhere [8]. Forty-five subjects who could not remember whether they had fallen during the preceding 12-month period or did not present any Kihon checklist result record were excluded from the analysis.

The data of three subjects who had mistakenly worn an insole with faulty sensor connections were excluded from the analysis. Additionally, the data of 14 subjects for whom it was impossible to detect at least three steps for each foot (c.f., “signal processing and data reduction”) were also withdrawn from the analysis. Finally, the data of 712 subjects (age: 71.3 ± 8.2 years, women: 505, men: 207, frail: 203, history of falling in the previous 12 months: 142) were used for the extraction of data features and statistical analysis.

### Signal processing and data reduction

**Fig. 2:**
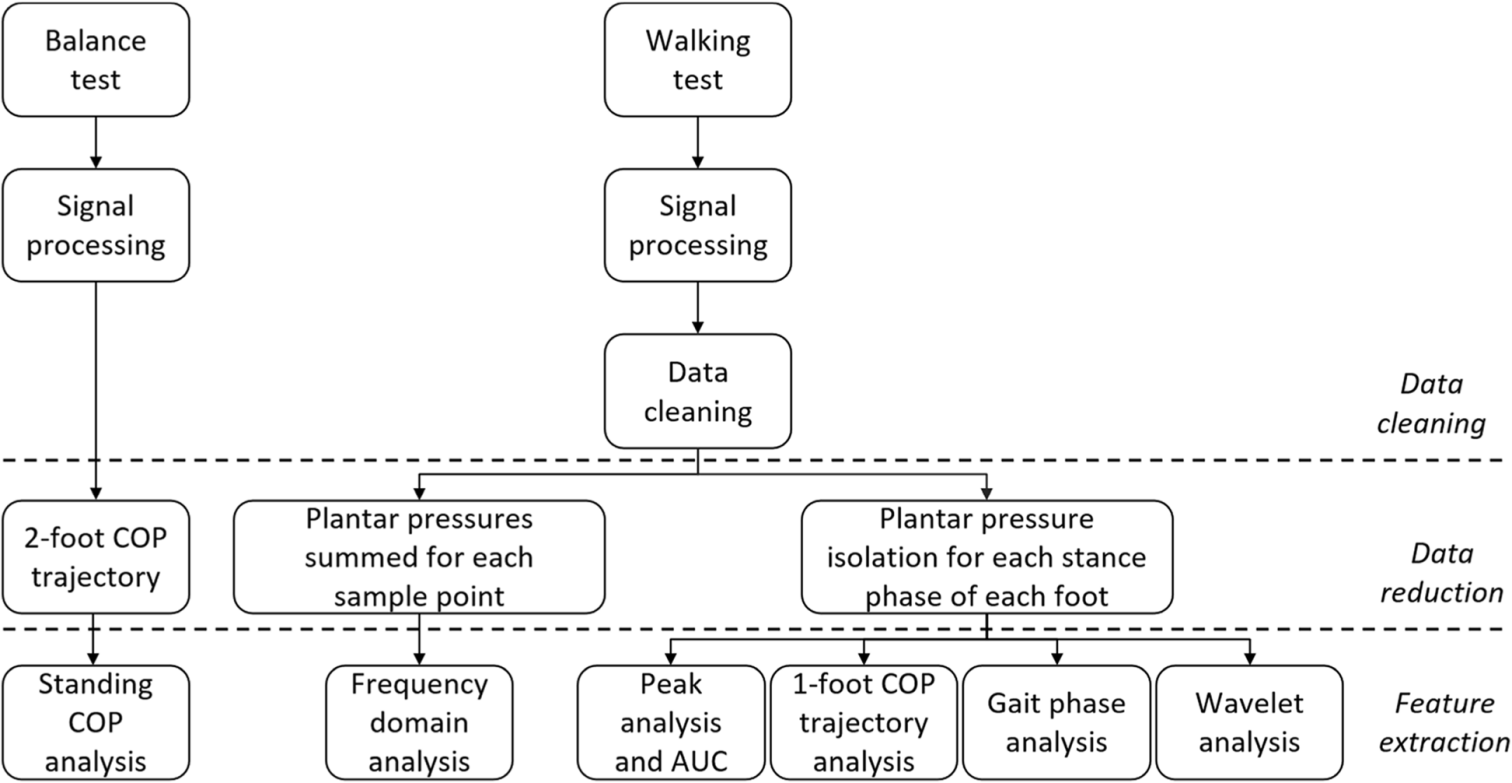
Overview of the data reduction process. AUC: area under the curve. COP: center of pressure

The raw data were converted into Newtons (N). Each data file contained 14 plantar pressure time series corresponding to the 7-sensor data for the left and right feet. The overall data reduction process is illustrated in Fig. 2. The plantar pressure data obtained during the balance test were used to compute the 2-foot COP excursion trajectory. The information was used to compute features related to “the standing COP analysis” (Fig. 2). Plantar pressure time-series data obtained from the walking tests were cleaned to focus on steps executed at a constant traveling speed. Depending on the subject, two to four steps corresponding to the acceleration and deceleration phases were manually removed from each 10 m test segment. Examples of the raw 2-foot plantar pressure time series are shown in Supplementary Material 3. Then, the time series was reduced in two different ways. First, the sum of the pressure outputs of the 14 pressure sensors was calculated at each sampling time. This new single time-series was used to compute the data features in the frequency domains (Fig. 2). Second, data corresponding to the stance phase of each foot was selected using an algorithm capable of detecting strike and lift events to build a new data set comprising the plantar pressure information for each isolated stance. This set of isolated steps (i.e., stance phases) was used for the computation of four categories of data features, extracted from the “peak analysis,” “1-foot COP trajectory analysis,” “gait phase analysis,” and “wavelet analysis.”

### Feature extraction

A total of 182 data features were extracted. They were adapted from parameters described in previous studies that used plantar pressure information to investigate the risk of falling [12, 22], explore gait alterations in various populations [20, 23, 31], or predict activities of daily life [29]. Features were derived from six different analyses of the data. In this section, the medial-lateral and anterior-posterior axes are denoted as x and y. One category of features was extracted from plantar pressure data collected during the standing balance test.

**Fig. 3:**
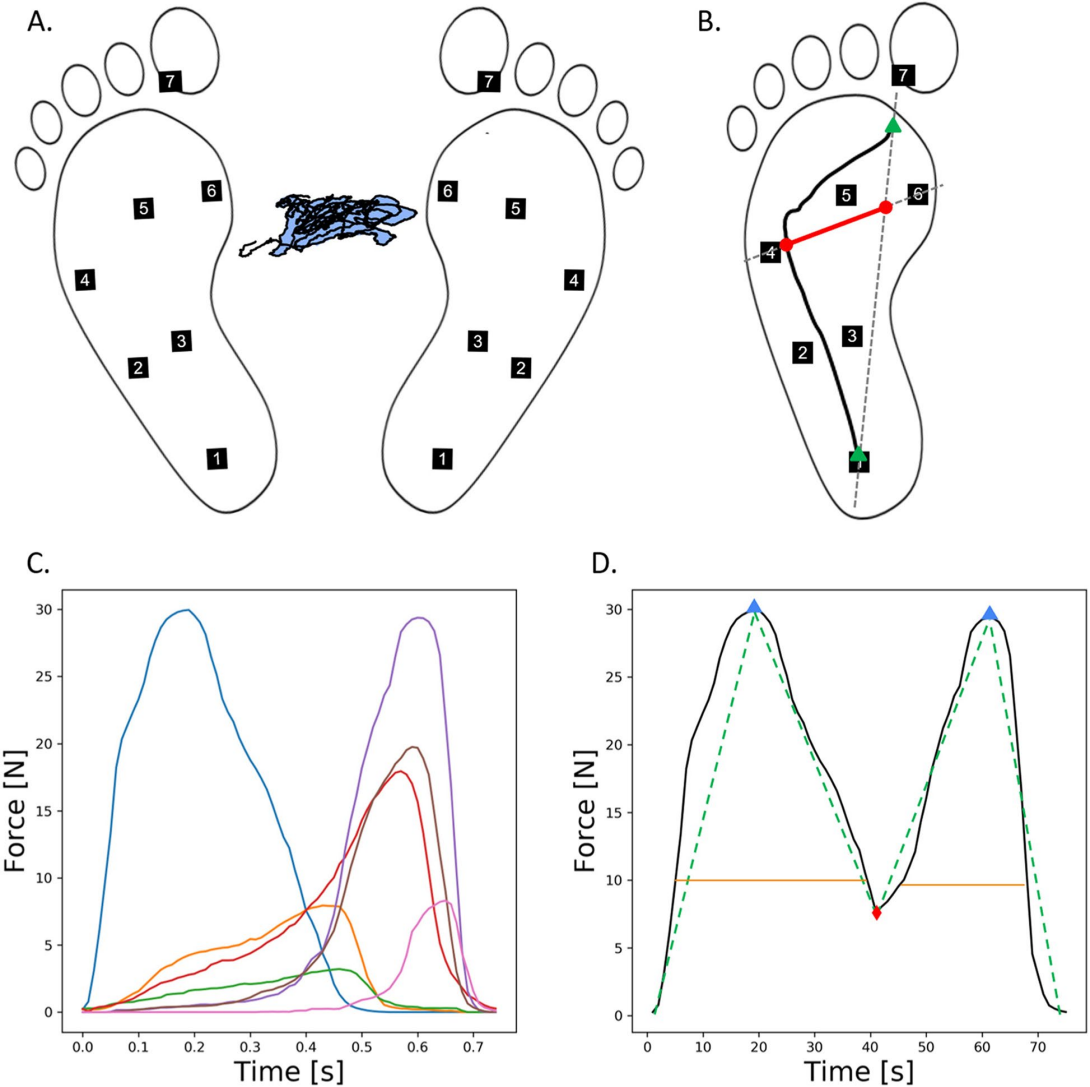
Illustration of some selected parameters computed during the data feature extraction process. **A**: Data features extracted from the Standing COP analysis. The black line illustrates the COP excursion trajectory. The light blue area illustrates the surface covered by the COP excursion trajectory. **B**: Variables used in 1-foot COP trajectory analysis. The black line illustrates the COP trajectory. The red line segment illustrates the 1-foot COP excursion. The numbered black squares indicate the virtual locations of the 7 sensors. The green triangle marks are the starting point and the endpoint of the COP trajectory. **C**: Example of plantar pressure time series for one isolated step obtained during the walking test. Plantar pressures treated for each isolated step are the raw material for the extraction of all time domain features in the following categories: “peak analysis and area under the curves”, “1-foot COP trajectory analysis”, “gait phase analysis” and “Wavelet analysis”. Blue: heel, orange: lateral midfoot, green: center of the midfoot, red: lateral forefoot, purple: center of the forefoot, brown: medial forefoot, pink: big toe. **D**: Variables used in the wavelet analysis (extracted from **C**). The black line corresponds to the envelope of the 7 sensors. The blue triangle illustrates the first and the second peaks typically observed during the stance phase. The red diamond illustrates the valley between the two peaks. The orange lines describe the peak widths, calculated at 30% of their magnitude. The green break lines correspond to the slopes on each side of the peaks. x: medial-lateral axis. y: anterior-posterior axis. In panel **A**, COP excursion distances were doubled on the x and y axes to increase readability

- Standing COP analysis. After computation of the COP excursion trajectory, the range of variations in x and y, the total length of the excursion trajectory, and the total surface covered by the COP excursion were calculated (Fig. 3A). This category includes four extracted features.

Five categories of features were extracted from plantar pressure data collected during the walking test.

- Frequency Domain Analysis. A fast Fourier transform was performed after the output of the 14 sensors was summed at each sampling point to integrate the temporal information on only one time series. The average power spectrum between 2 and 10 Hz, the standard deviation of power spectrum between 2 and 10 Hz, power density, and mean frequency between 2 and 10 Hz were computed. This category includes four extracted features.
- Peak analysis and area under the curves. First, three parameters were extracted for each isolated step and each sensor. These parameters include 1) the maximum pressure and 2) the time at which this maximum pressure occurred relative to the total stance time. In addition, each isolated step was resampled on a 100-point long band to obtain time-standardized data. 3) The area under the pressure curve was extracted for each isolated step and for each sensor. Second, the four following data features were calculated for each trial and each of the three parameters: 1) the average of all the left foot steps, 2) the standard deviation of all the left foot isolated steps, 3) the standard deviation of all isolated steps of both feet, and 4) the left and right foot average difference. Thus, four features were computed for three parameters and seven sensors, resulting in 84 extracted features in this category.
- 1-foot COP trajectory analysis. The COP trajectory was computed for each stance phase of each isolated step. First, the following 13 parameters were extracted: the minimum and maximum values on x and y, x and y coordinates at the double to single stance and single to double stance points, x coordinates of COP at the y coordinates of the center midfoot and center forefoot sensors, respectively; the range of variations on x and y; and the center of pressure excursion index, calculated as the ratio of COP trajectory excursion on the distance between the lateral and medial forefoot sensors (Fig. 3B). Second, the four following data features were calculated for each trial and each of the 13 parameters: 1) the average of all the left foot isolated steps, 2) the standard deviation of all the left foot isolated steps, 3) the standard deviation of all isolated steps of both feet, and 4) the left and right foot average difference. This category included 52 extracted features.
- Gait phase analysis. The following two parameters were computed for each isolated step: 1) stance phase duration and 2) percentage of double support duration relative to the whole stance phase. Then, the following four features were extracted for each trial and each of the two parameters: 1) average of all the steps from the left foot, 2) standard deviation of all the steps from the left foot, 3) the standard deviation of all isolated steps of both feet, and 4) the left and right foot average difference. This subcategory includes a total of 8 extracted features.
- Wavelet analysis. For each stance phase of each isolated step, the envelope of the 7-sensor of the left foot was computed [31]. This category of features is based on the characteristics of the two waves, which characterize the plantar pressure pattern during the stance phase (Fig. 3C and D). First, the following 15 parameters were computed: 1) the distance between the first and second peaks, 2) height of the first peak, 3) height of the second peak, 4) height of valley, 5) the difference between the heights of the peaks, 6) ratio of the height of the first peak to one of the valleys, 7) ratio of the height of the second peak to one of the valleys, 8) difference between these two ratios, 9) width of the first peak, 10) width of second peaks, 11) difference between these two widths, 12) slope rate from the starting point of the stance phase to the first peak, 13) slope rate from the first peak to valley, 14) slope rate from valley to the second peak, 15) slope rate from the second peak to the endpoint of stance phase. Second, the average and standard deviation of all the steps from the left foot were calculated, resulting in the extraction of two features for 15 parameters. This category included 30 extracted features.

At the end of the data reduction and feature extraction processes, each subject was associated with one data point of 182 dimensions.

### Classification using random forests

Random forest models were used to classify the frailty state and fall history. The 182 features extracted from the plantar pressure data were used as the input. The machine-learning analysis was implemented using the Python scikit-learn and imbalanced-learn modules [32]. Random forests are ensemble models that rely on large collections of independent decision trees to increase predictive performance compared to standalone decision trees. All trees are trained in parallel over a random bagged subset of the data, that is, a set of data of the same size as the original set but where data points may appear multiple times or be absent. Bagging adds independent biases in individual classification trees, thus preventing overfitting. The overall prediction is obtained through the majority vote of individual decision trees. In the present study, the number of non-frail subjects was larger than that of frail subjects. Similarly, the number of subjects without a history of falling was larger than the number of subjects with a history of falling. To avoid issues related to imbalanced datasets, such as the random forest performing poorly on minority classes due to overtraining on the majority class, each tree of the forest was built with a balanced subset of samples, using the balanced random forest down-sampling algorithm described elsewhere [33].

Models were built for the whole population or some selected subgroups relative to age (≥65, 60–69, 70–74, ≥75 years old), sex (women, men), and frailty state (Table 1). Finally, additional analyses were conducted with data features extracted from the standing balance test only. Models showed low classification performances (average balanced accuracy: 0.57 ± 0.05, weighted F1-score: 0.556 ± 0.034) Detailed results are not shown.

**Table 1:** Number of subjects for each class (frail vs. non-frail and falling event vs. no falling event) in each subgroup

	Frail	Non-frail	Total	Falling event	No falling event	Total
Whole population	203	509	712	142	570	712
Age-group:						
≥65 years	193	392	585	126	459	585
65–69 years	22	176	198	35	163	198
70–74 years	42	111	153	35	118	153
≥ 75 years	129	105	234	56	178	234
Total	193	392	585	126	459	585
Sex:						
Male (≥ 65)	50	126	176	88	88	176
Female (≥ 65)	143	266	409	38	371	409
Total	193	392	585	126	459	585
Frailty state:						
Frail	–	–	–	52	151	203
Non-frail	–	–	–	90	419	509
Total	–	–	–	142	570	712

The training procedure was set so that each forest model was composed of 200 decision trees. Each tree is built by successfully splitting its nodes until the Gini impurity score equals zero until all data points in the leaf nodes correspond to the same class.

Then, the models were validated using a 5-fold cross-validation procedure. Data were split into five equal subsamples, with each subsample retained once as the validation data to test the model constructed with the other four subsamples. The results of the five tests were averaged to determine the overall performance of the model. The training and validation procedures were repeated 100 times with different random subsample splits. The balanced accuracy and weighted F1-score were calculated to assess the performance of the models. Detailed confusion matrices are presented for the models built on the whole population. Alternatively, random forest models were constructed and tested using a nested cross-validation procedure similar to that described elsewhere [34]. The results are similar and are shown in Supplementary Material 4.

### Feature contributions

Additional analyses were conducted to identify the most informative data features in random forest models built to classify frail and non-frail subjects. The “feature importance” tool integrated with the Python scikit-learn module was used to perform this operation [32]. Briefly, the importance of features appearing in a tree is evaluated according to the subsequent decrease in sample impurities. The mean decrease in impurity is calculated across the forest, and features are ranked according to this score, that is, according to the capacity of features to make the model converge quickly toward one class. Then, the ranks of each feature across the 100 subsample splits were averaged to evaluate the overall capacity of those features to influence the classification of frail and non-frail subjects across the range of subsamples and subgroup configurations tested in the present study.

### Classification using logistic regressions

Finally, multi variable logistic regressions models were also used to classify the frailty state and fall history. The variables used in the models corresponded to the 182 features extracted from the plantar pressure data. Similar to what was done for the random forest classifiers, logistic regression models were validated using a 5-fold cross-validation procedure and the training and validation procedures were repeated 100 times with different random subsample splits. The feature importance was evaluated by comparing the regression coefficients for each variable. The analysis was implemented using the Python scikit-learn library. The logistic regression models showed lower classification performances compared to random forest classifiers, which is consistent with previous observations related to the use of random forest classifiers vs. logistic regression methods in clinical sciences [35]. Results of the logistic models and their interpretation are shown in Supplementary Material 5.

## Results

### Classifying frailty

**Fig. 4:**
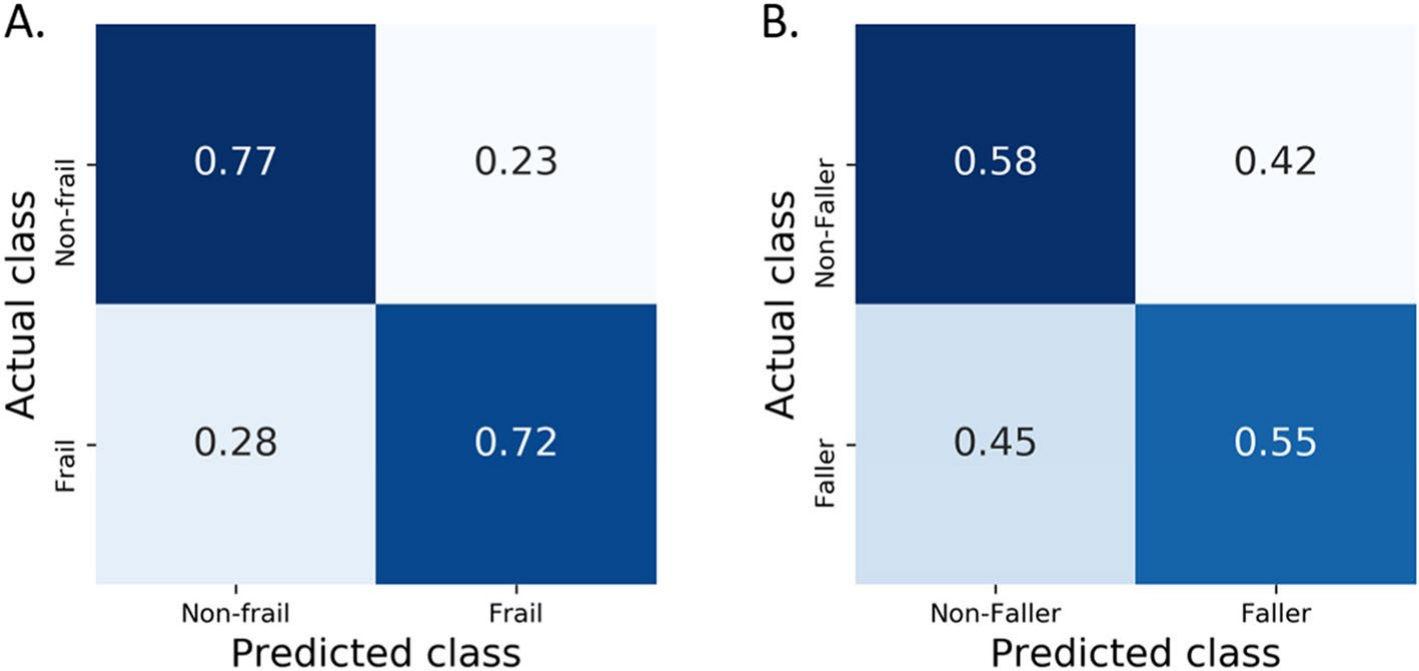
Performance of the frailty state and falling history classifiers for the whole population presented as confusion matrices. **A**: frailty state. **B**: falling history

As shown in Table 2, the random forest classifiers showed an average balanced accuracy of 0.75 ± 0.04 and an average weighted F1-score of 0.77 ± 0.03 for the recognition of frail vs. non-frail subjects. More specifically, as shown in Fig. 4A, 72% of subjects evaluated as frail by the Kihon Checklists were also classified by the plantar pressure data-fed random forest models. Seventy-seven percent of subjects who had been evaluated as non-frail by the same checklist were also classified as non-frail by the models.

**Table 2:** Summary of balanced accuracies and weighted F1-scores of random forest models

	Frailty predictions		Fall predictions	
	Accuracy	F1-score	Accuracy	F1-score
Whole population (*N* = 712)	0.75 ± 0.04	0.77 ± 0.03	0.57 ± 0.05	0.62 ± 0.04
Age-group:				
≥65 years (*N* = 585)	0.76 ± 0.04	0.77 ± 0.04	0.55 ± 0.05	0.59 ± 0.04
65–69 years (*N* = 198)	0.68 ± 0.11	0.78 ± 0.05	0.60 ± 0.10	0.67 ± 0.06
70–74 years (*N* = 153)	0.68 ± 0.09	0.70 ± 0.07	0.49 ± 0.10	0.53 ± 0.08
≥ 75 years (*N* = 234)	0.71 ± 0.06	0.71 ± 0.06	0.54 ± 0.08	0.58 ± 0.07
Sex:				
Women (≥ 65 years, *N* = 409)	0.72 ± 0.04	0.74 ± 0.04	0.56 ± 0.05	0.61 ± 0.04
Men (≥ 65 years, *N* = 176)	0.78 ± 0.07	0.79 ± 0.05	0.49 ± 0.09	0.56 ± 0.07
Frail state:				
Frail (*N* = 203)			0.49 ± 0.08	0.53 ± 0.07
Non-frail (*N* = 509)			0.58 ± 0.06	0.64 ± 0.04

Results are presented as mean ± standard deviation

Regarding the age-group analyses, the results were slightly better when considering only people over 65 years of age (accuracy: 0.76 ± 0.04; F1-score: 0.77 ± 0.04). On the other hand, models built for the 65–69 and 70–74 years old subgroups showed lower performance (accuracies: ≃ 0.68). Regarding sex groups, accuracies and F1-scores were higher when considering men only (0.78 ± 0.07 and 0.79 ± 0.05, respectively).

**Fig. 5:**
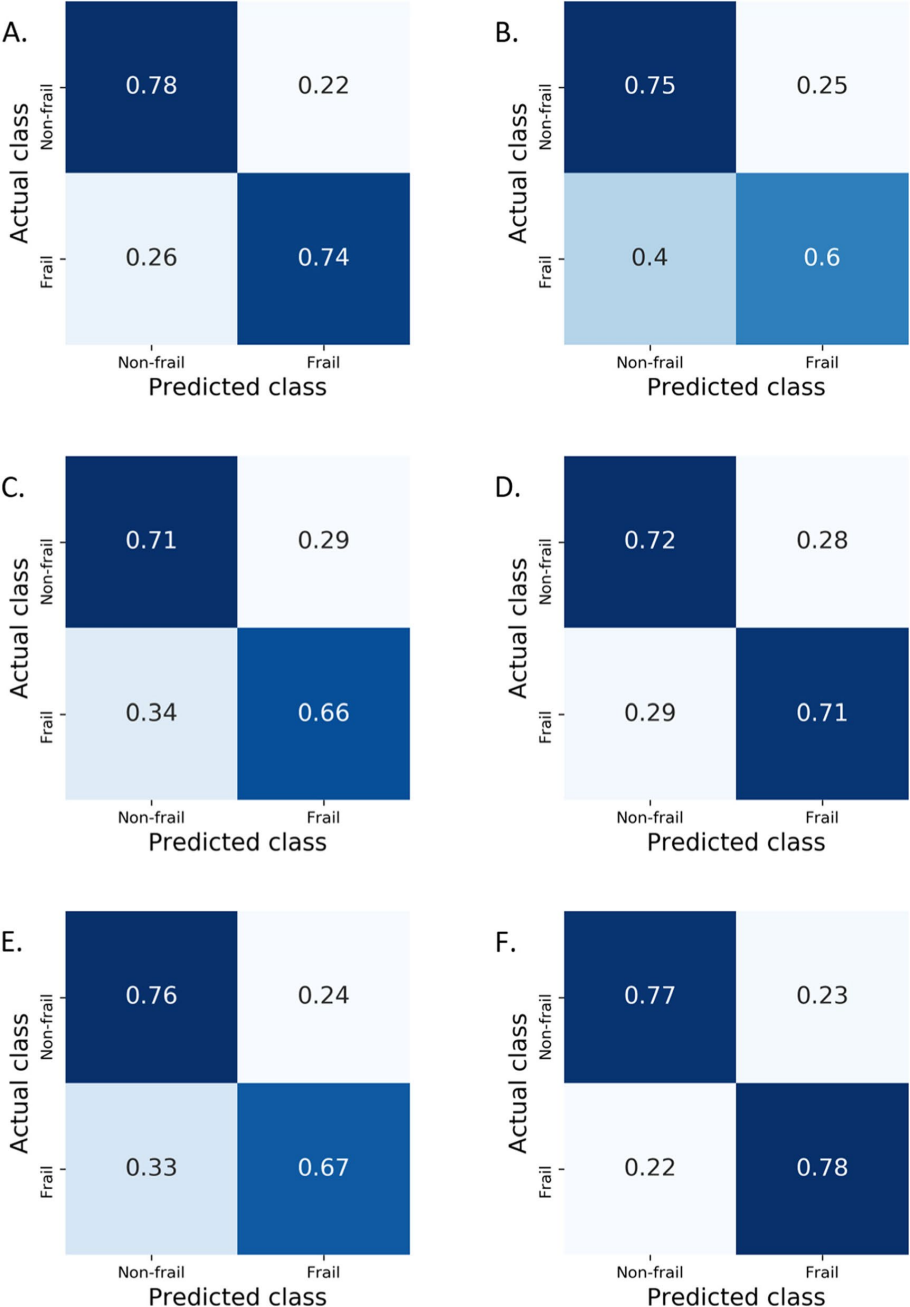
Performance of the frailty state classifiers presented as confusion matrices for all the subgroups. **A**: aged ≥65 years old. **B**: aged between 60 and 69 years old. **C**: aged between 70 and 74 years old. **D**: aged ≥75 years old. **E**: women only (≥ 65 years). **F**: men only (≥ 65 years)

The detailed outcomes of random forest models for classifying frail versus non-frail subjects in each subgroup are shown in the confusion matrices in Fig. 5.

### Classifying fallers

The random forest classifiers showed an average balanced accuracy of 0.57 ± 0.05 and an average weighted F1-score of 0.62 ± 0.04 for classifying fallers vs. non-fallers. As shown in Fig. 4B, 55% of subjects with a history of falling in the year preceding the test were classified by the random forest models trained on plantar pressure data. Fifty-seven percent of subjects who did not present any history of falling in the previous year were correctly classified. Regarding the age-group analyses, the best performances were obtained for the 65–69 years old subgroup (accuracies: 0.60 ± 0.10; F1-score: 0.67 ± 0.06). As shown in Table 2, lower performance was observed in the women- or men-only subgroups. Accuracies of 0.49 ± 0.08 and 0.58 ± 0.06 were found for the frail and non-frail population models, respectively (F1-score: 0.53 ± 0.07 and 0.64 ± 0.04).

### Feature contributions

Considering models built for the whole population, the ten most informative features were: 1) the average of the ratios of the height of the second peak to the height of the valley point [Wavelet analysis], 2) the standard deviation of the ratios of the height of the first peak to the height of the valley point [Wavelet analysis], 3) the average of the ratios of the height of the first peak to the height of the valley point [Wavelet analysis], 4) the average of stance phase durations [Gait phase analysis], 5) the standard deviation of the ratio of the height of the second peak to the height of the valley point [Wavelet analysis], 6) the average of the slope rate from the starting point of the stance phase to the first peak [Wavelet analysis], 7) the average of maximum pressure of sensor 1 [Peak analysis and area under the curves], 8) the average of the area under the curve of sensor 3 [Peak analysis and area under the curves], 9) the average of the height of the second peak [Wavelet analysis], and 10) the average of the time when the maximum pressure occurred relative to the total stance time for sensor 1 [Peak analysis and area under the curves].

Considering all the subgroups, 17 additional features were ranked among the ten most informative features (Table 3). Three variables were ranked among the 10 most important features in all analyses (i.e., whole population analysis and all subgroup analyses). In addition, 12 variables were ranked among the 10 most important features in more than one analysis. Features extracted from the wavelet analysis accounted for 10 of the 27 identified important features and 6 of the top-10 important features. Three features from the category “peak analysis and area under the curves” ranked among the top-10 important features. The remaining results regarding the contributions of the most essential features are described in Table 3.

**Table 3:** Important features for classifying frailty in the whole population and relative to each subgroup

	Whole population models	≥ 65 years old models	65–69 years old models	70–74 years old models	≥ 75 years old models	≥ 65 women models	≥ 65 men models
[Wavelet analysis] Ratio of height of peak 2 to height of valley (average of left foot)	O	O	O	O	O	O	O
[Wavelet analysis] Ratio of height of peak 1 to height of valley (SD of left foot)	O	O	O	O	O	O	O
[Wavelet analysis] Ratio of height of peak 1 to height of valley (average of left foot)	O	O	O	O	O	O	O
[Gait phase analysis] Stance phase duration (average of left foot)	O	O	O		O	O	O
[Wavelet analysis] Ratio of height of peak 2 to height of valley (SD of left foot)	O	O	O	O	O	O	
[Wavelet analysis] Slope rate from the starting point to peak 1 (average of left foot)	O	O					O
[Peak analysis and AUC] Maximum pressure of heel sensor (average of left foot)	O	O				O	
[Peak analysis and AUC] AUC of center midfoot sensor (average of left foot)	O	O	O	O		O	
[Wavelet analysis] Height of peak 2 (average of left foot)	O					O	O
[Peak analysis and AUC] Time when maximum pressure of heel sensor occurred (average of left foot)	O	O			O		
[Gait phase analysis] Percentage of double support phase duration (average of left foot)		O	O			O	
[1-foot COP trajectory analysis] Y coordinate of double-to-single support phase transition (average of left foot)			O				
[Wavelet analysis] Height of peak 1 (SD of left foot)			O				
[1-foot COP trajectory analysis] Minimal value on y axis (SD of left foot)			O				
[Peak analysis and AUC] Maximum pressure of center midfoot sensor (average of left foot)				O			
[Peak analysis and AUC] Maximum pressure of center midfoot sensor (SD of both feet)				O			
[Peak analysis and AUC] Maximum pressure of center midfoot sensor (Difference of two feet)				O			
[Peak analysis and AUC] AUC of center midfoot sensor (SD of both feet)				O			
[Peak analysis and AUC] AUC of center midfoot sensor (average of left foot)				O			
[1-foot COP trajectory analysis] CPEI (SD of left foot)					O		
[1-foot COP trajectory analysis] X at y coordinate of center midfoot sensor (SD of left foot)					O		
[Peak analysis and AUC] Maximum pressure of center forefoot sensor (SD of both feet)					O		
[Peak analysis and AUC] Time when maximum pressure of center midfoot sensor occurred (average of left foot)					O		O
[Wavelet analysis] Slope rate of peak 2 to the endpoint (average of left foot)						O	
[Wavelet analysis] Slope rate from valley to peak 2 (average of left foot)							O
[Frequency domain analysis] SD of power spectrum							O
[Wavelet analysis] Distance between peak 1 and peak 2 (average of left foot)							O

For the whole population models, features are listed from top to bottom in the same order as they ranked (see the top 10 rows). Then, new important features found in the 1) subjects aged ≥65 years old, 2) subjects aged between 60 and 69 years old, 3) subjects aged between 70 and 74 years old, 4) subjects aged ≥75 years old, 5) women aged≥65 years, and 6) men aged ≥65 years analyses appear in the 17 remaining rows

## Discussion

This study investigated the feasibility of using plantar pressure data to identify frail people and predict fall events in the elderly. Over 700 senior people performed a balance standing test and a 20 m walking trial while wearing a 7-sensor plantar pressure measurement insole. One-hundred-eighty-two features were extracted from the collected plantar pressure data. Random forest models were built to identify subjects with a frail state or a recent history of falling. The overall balanced accuracy for the recognition of frail subjects was 0.75 ± 0.04 (F1-score: 0.77 ± 0.03). The overall balanced accuracy for classifying subjects with a recent history of falling was 0.57 ± 0.05 (F1-score: 0.77 ± 0.03). The classification of subjects relative to their frailty state primarily relied on features extracted from the plantar pressure series collected during the walking test. In particular, the classifiers frequently used features related to plantar pressure peaks, i.e., the “Wavelet analysis” and “Peak analysis and AUC” categories. In the future, plantar pressure data processed with random forest algorithms might be of interest to support the detection of gait-related frailty patterns. Further research works are necessary to understand how the tools used in the present study could complement the existing evaluation methods. In the present study, these tools were ineffective in classifying subjects according to their history of falling.

### Plantar pressure measurement for classifying frail individuals and fallers

Studies proposing new assessment methods for frailty in senior people are regularly published [7]. The use of technology allows for more objective evaluations and is therefore attractive to clinicians. To date, several studies have successfully combined the use of inertial sensors with statistical classification techniques [11, 12, 15]. Only one study has tried to use plantar pressure to distinguish frail people from healthy individuals [12]. In a group of 186 senior people, Chkeir et al. extracted four parameters from the vertical ground reaction force analysis and COP position when stepping on a bathroom scale composed of a 4-sensor force platform. Unlike the present study, measurements were completed in static conditions only. The authors found statistical differences between healthy, pre-frail, and frail individuals but did not use machine learning techniques to develop classifying models.

The present study is the first to combine plantar pressure measurements with machine learning techniques to classify frail and healthy senior people. Among studies aiming to introduce new technology for assessing frailty, this is also the second study to test a large sample of over 700 senior people [13]. The accuracy score of 0.75 ± 0.04 may not be as high as some previous studies that used accelerometer sensors and functional tests [15, 36]. In one study aiming at classifying pre-frail and healthy subjects in a group of 124 elderly people, Greene et al. [15] reported accuracy scores of 0.84 (F1-score: 0.83) and 0.94 (F1-score: 0.94) in women and men, respectively. They collected kinematic data using a network of inertial sensors attached to different parts of the body during the completion of established clinical instruments, such as TUG, sit-to-stand, and standing balance tests. In another study consisting in classifying frail and robust subjects in a group of 309 elderly people (training sample:160, test sample: 149), Chang et al. (2013) reported an accuracy score of 0.83 (F1-scores: 0.81) [36]. They used a complex experimental set-up combining sensor units attached to several selected pieces of home furniture, again in conjunction with functional tests. They also input the data obtained from digital questionnaires surveying subjects abilities to perform activities of daily living. In contrast, the plantar pressure data used in the present study were obtained during a simple 45 s standing test and two 10 m walking trial segments; these data were obtained using a single easy-to-use instrument, i.e., the plantar pressure measurement insole, not complex multi-sensing systems used in conjunction with clinical instruments or functional tests, as the ones proposed in the above-mentioned studies [15, 36]. Perhaps, plantar pressure data obtained in the course of a TUG, sit-to-stand test, or any other challenging situation (e.g., dual tasks, etc.) would also result in higher accuracy scores. Future studies are necessary to verify this hypothesis and to understand whether the combination of features extracted from inertial sensors and in-shoe plantar pressure measurements would yield better results for identifying frail people.

Interestingly, higher performances have been noted for men than women (0.78 ± 0.07 vs. 0.72 ± 0.04). These observations are similar to those of studies that used inertial sensors and may be explained by some women-specific gait characteristics [15, 37]. Walking speed, step length, and step width were found to be lower in aging women than in their male counterparts, which points to the necessity of developing specific models for each population.

In the present study, models developed for identifying people with a recent history of falling did not show satisfactory results. The best performance was as low as 0.60 ± 0.10, only for the 65–69 age group. Further studies are needed to clarify whether models using plantar pressure data obtained in functional tests, rather than simple standing balance tests and 20 m walking trials, could yield better predictions. Plantar pressure data could also be collected in free-living conditions to try detecting near-fall events (i.e., slips, trips, missteps), the frequency of which has been shown to be associated with the risk of future actual falls [38]. The question of the adequacy of the extracted features may also be considered. While COP-related features have already shown statistical relationships with falling events in at least on previous study [22], features describing one-dimensional ground reaction forces had never been suggested in the literature and may not have the same prediction capabilities as for the frailty state prediction models. Finally, the fall history recall questionnaire used in the present study did not allow distinguishing events caused by intrinsic physical factors from the ones caused by extrinsic/environmental factors. Factors falling into the second category may not involve any physical change that could be captured by the 7-sensor plantar pressure measurement insole device used in the present study.

### Plantar pressure measurements and feature extraction

Investigating the features that contribute the most in random forest classifier models may provide early insight into the physical changes that could be important for the early detection of frailty patterns. Considering previous observations on the age-related changes in walking COP trajectories and the call for using walking COP measurements for the evaluation of gait stability and postural control abilities, features extracted from COP excursion and trajectories during standing and walking trials were expected to rank among the most important features for the detection of frail individuals in the present study [23]. Instead, features providing the most valuable information to the random forest models were those related to the ground reaction force (Fig. 3C and D). Ratios of the height of peaks to the height of the valley, alongside several other parameters from the wavelet analysis, were among the most contributive features. Such parameters are associated with moving the center of gravity efficiently during the gait stance phase [39]. While the sharpness of the ground reaction force wave is closely related to walking speed in healthy subjects, alterations of this wave during walking trials have also been linked with pathologies of the lower limbs. For instance, Kotti et al. successfully used similar parameters to identify knee osteoarthritis patients [31]. Moreover, other parameters from the sensor-specific peak and AUC analysis have also been identified among the most contributive ones. Features related to the heel and center midfoot sensors are especially well represented, indicating that features reflecting the ability to sustain landing load at the beginning of the stance phase may also be considered early frailty indicators.

Interestingly, all the 27 important features identified in the present study emanate from plantar pressure data collected during the walking trial, pointing to the limit of the force plate for the evaluation of frailty and the necessity to develop systems capable of performing measurements during ambulatory trials. Moreover, random forest classifiers built with data features extracted from the standing balance test only showed a lower accuracy (balanced accuracy: 0.57 ± 0.05, weighted F1-score: 0.56 ± 0.04, detailed data not shown).

### Smart insole for the early detection of frailty patterns

The objective evaluation of frailty state and falling risk in senior people remains a critical contemporary challenge in the health science field. The assessment of plantar pressures could provide crucial pieces of information, more specifically for the evaluation of the physical dimension of frailty. Indeed, aging-related gait alteration is associated with some loss of strength or with the development of sarcopenia [40]. The early evaluation of parameters that inform on the physical dimension of frailty would enable tailoring appropriate interventions early in the aging process. Plantar pressure measurements have been linked with promising preliminary observations in the past. Cheap and wireless smart-insoles similar to the one used in the present study could overcome some of the practical issues related to the use of force plates, especially when measurements are carried out during walking trials [20–23].

In addition, at the dawn of the IoT era, it is certainly possible to design smart shoe devices that can systematically collect plantar pressure data during daily life walking segments and monitor changes in COP trajectories and ground force reaction waves over several years. Considering the relatively good frailty classification accuracies produced in the present study with data extracted from a minimal number of steps, it is possible to expect higher scores with longitudinal approaches. Moreover, longitudinal monitoring of plantar pressure data in free-living conditions through smart shoe devices should not be restricted to walking segments only. Physical behavior recognition using 7-sensor plantar pressure measurement insole devices is feasible [29]. Therefore, it would be possible to isolate sit-to-stand events that naturally occur during the daily life of older adults and analyze the plantar pressure data to detect deviations in frailty patterns. Piau et al. tested the feasibility of using smart shoes to collect behavioral information in free-living conditions and for long periods of time. They observed a high level of acceptance in senior people [41]. Their smart insole device could track the number of steps, walking distance, gait speed, and active walking duration, but no functional evaluation of the participants was performed. Therefore, longitudinal and prospective studies are needed before stating on the relevance of the smart shoe approach for the individualized surveillance of gait and balance function alteration and the early detection of frailty patterns. These studies should consider how to use this new approach concomitantly to the existing methods in order to properly evaluate how they can complement them by bringing new or earlier information to the clinicians.

### Limitations and strengths

One limitation of the current study is related to the imbalanced nature of the dataset. Twenty-nine percent of the subjects were defined as frail using the Kihon checklist, and only 20% of the subjects declared having experienced at least one fall event in the year preceding the measurements, resulting in a limited amount of data to train the algorithm with regard to the characteristics of these two groups. Consequently, one cannot rule out that the lower performance observed with the faller classification algorithms could be a consequence of the limited available data rather than irrefutable evidence that the 7-sensor plantar pressure measurement system proposed in the present study is unsuitable for the identification of fallers. In some subgroups, the ratio of frail subjects to non-frail subjects was extremely low. For example, no more than 11% of people aged between 65 and 69 years old were categorized as frail by the Kihon checklist, resulting in classifiers having lower performance in this age group (accuracy: 0.68 ± 0.11, F1-score 0.78 ± 0.05). Steps were taken to address this limitation. First, a large number of people (774) were recruited. The minority class could include enough samples and a variety of postural and gait patterns representative of the senior Japanese population. The study included 712 participants, which means that data from over 110 and 160 participants, for the faller and frailty analyses, respectively, were available for training the whole population models. To date, only one other study has tested the effect of wearable technology for assessing frailty or the risk of falling on such a high number of subjects [7, 12]. The 30–70 ratio between frail and non-frail people found in the present study is 7.4%. This is higher than the reported estimated prevalence of frailty in senior Japanese people [42]. This higher figure may be explained by the fact that healthier individuals are less present in the spaces through which the subjects were recruited (i.e., health and welfare administration and healthcare provider company) or less interested in having this type of postural and gait assessment. Second, the majority class has been under-sampled according to the method described elsewhere [33], in order to avoid 1) classifiers performing poorly on minority classes due to overtraining in the majority class and 2) overfitted outcomes that come from the paucity of information in the minority class.

Another major limitation relates to the standards adopted in this study to identify frailty. In the absence of the gold standard method, the Kihon Checklist was used to determine frailty status. The method is widely used in Japan and has been described as a valid frailty prediction tool in several reports [43, 44]. However, many tests are available to predict frailty. Some authors have suggested that results could vary widely, especially between self-administrated methods such as the Kihon checklist and tests administered by nurses or physicians [45]. Therefore, it is possible that the accuracy score of the present study could have been different, either increased or decreased, if another frailty assessment tool had been used as a reference instead of the Kihon checklist. In the future, new objective assessment methods, such as the one proposed in the present study, should be tested against a broader panel of frailty assessment methods to strengthen the interpretability of the results.

Another limitation of the present study is the non-inclusion of variables related to the medical history of participants in the predicting models. Indeed, a combination of plantar pressure data and medical information could strengthen accuracy scores for either falling history or frailty state predictions. However, building such kind of models would not only have required a systematic collection of medical history, but also an even larger group of participants to have enough individuals per medical condition so that the learning algorithms can identify patterns.

Finally, the present protocol does not allow excluding the presence of inaccuracies for the falling history parameter, which may have negatively impacted the accuracy scores. Future studies should include a more robust protocol for the collection of information related to falling events, be they recalled data or prospective protocols.

## Conclusion

Plantar pressure data collected with a simple 7-sensor insole during a 45 s standing balance test and a 20 m walking trial have been successfully used to identify frail people. Signal features extracted from the wavelet analysis, reflecting body swing during the stance phase, were identified as the most important contributors to the classifier models considered in the present study. In the absence of a gold standard for evaluating frailty, this new objective method could be used to help detect frailty in older adults. At the dawn of the IoT era, plantar pressures could be collected continuously using a 7-sensor insole similar to that used in the present study but with 5G capabilities. This would allow the identification of early markers of frailty able to complement the information already available to the health professionals through the existing methods.

In the present work, attempts to classify people with a history of falling were unsuccessful. However, prospective studies need to be conducted. This would also allow further exploration of the feasibility of using plantar pressures to detect potential fallers.

## Supplementary Information

**Additional file 1.**

**Additional file 2.**

**Additional file 3.**

**Additional file 4.**

**Additional file 5.**

**Additional file 6.**

## Data Availability

All data and codes used in the random forest classifier analysis are included in this published article and its supplementary information files.
