# Correction: Random forest algorithms to classify frailty and falling history in seniors using plantar pressure measurement insoles: a large-scale feasibility study

**DOI:** 10.1186/s12877-022-03514-5

**Published:** 2022-12-08

**Authors:** Emi Anzai, Dian Ren, Leo Cazenille, Nathanael Aubert-Kato, Julien Tripette, Yuji Ohta

**Affiliations:** 1 Faculty of Engineering, Nara Women’s University, Nara, Japan; 2 Department of Cooperative Major in Human Centered Engineering, Graduate School of Humanities and Sciences, Ochanomizu University, Tokyo, Japan; 3 Department of Information Sciences, Ochanomizu University, Tokyo, Japan; 4 Center for Interdisciplinary AI and Data Science, Ochanomizu University, Tokyo, Japan; 5 Department of Human-Environmental Science, Faculty of Human Life and Environmental Sciences, Ochanomizu University, Tokyo, Japan; 6 Faculty of Core Research Natural Science Division, Ochanomizu University, Tokyo, Japan

**Correction To: BMC Geriatrics (2022) 22:746**

10.1186/s12877-022-03425-5

After publication of this article [1], the authors reported that affiliation 4 was incorrectly given as ‘Comprehensive Research Building, 6th floor, room 613, Bunkyo, Tokyo 112-8610, Japan’, but should have been ‘Center for Interdisciplinary AI and Data Science, Ochanomizu University, Tokyo, Japan’.

The original article [1] has been corrected.
