# Supplementary material for: Random forest algorithms to classify frailty and falling history in seniors using plantar pressure measurement insoles: a large-scale feasibility study
Source: BMC Geriatr. 2022 Sep 12;22:746. doi: 10.1186/s12877-022-03425-5 (PMC9469527; doi:10.1186/s12877-022-03425-5)
Supplement: Supplementary file 1 — Additional file 1. [file 12877_2022_3425_MOESM1_ESM.docx]

**Supplementary Material 1.**

**Kihon Checklist**

BMI, body mass index. Adapted from Satake et al. Validity of the Kihon Checklist for assessing frailty status. Geriatr Gerontol Int. 2016. Items 1-15 were used in the present study to evaluate frailty. The Kihon checklist was originally developed to identify senior individuals at risk of requiring frailty-related nursing care. The prevailing criteria were as follows: ≥10 for questions 1–20 (overall frailty), ≥3 for questions 6–10 (physical function domain), 2 for questions 11 and 12 (nutritional domain), or ≥2 for questions 13–15 (oral domain). Meeting one of these four criteria indicate that the respondent need frailty-related nursing care or prevention intervention care. Further observations made elsewhere (Fukutomi et al. Geriatrics Gerontology 2014, Satake et al. Geriatrics Gerontology International 2015) indicate a good alignment between the Kihon checklist outcome and the ones of other screening modalities.

References:

Fukutomi E, Okumiya K, Wada T, Sakamoto R, Ishimoto Y, Kimura Y, Chen WL, Imai H, Kasahara Y, Fujisawa M, Otsuka K, Matsubayashi K. Relationships between each category of 25-item frailty risk assessment (Kihon Checklist) and newly certified older adults under Long-Term Care Insurance: A 24-month follow-up study in a rural community in Japan. Geriatr Gerontol Int. 2015 Jul;15(7):864-71. doi: 10.1111/ggi.12360. Epub 2014 Oct 15. PMID: 25316532.

Satake S, Senda K, Hong YJ, Miura H, Endo H, Sakurai T, Kondo I, Toba K. Validity of the Kihon Checklist for assessing frailty status. Geriatr Gerontol Int. 2016 Jun;16(6):709-15. doi: 10.1111/ggi.12543. Epub 2015 Jul 14. PMID: 26171645.
