# Supplementary material for: Random forest algorithms to classify frailty and falling history in seniors using plantar pressure measurement insoles: a large-scale feasibility study
Source: BMC Geriatr. 2022 Sep 12;22:746. doi: 10.1186/s12877-022-03425-5 (PMC9469527; doi:10.1186/s12877-022-03425-5)
Supplement: Supplementary file 2 — Additional file 2. [file 12877_2022_3425_MOESM2_ESM.docx]

**Supplementary Material 2.**

*Repeatability test #1*: 5N incremental load applied 10 times to one sensor (range: 20-50N).

**Output of one single sensor for an incremental load**

| **Loading times** | **20N** | **25N** | **30N** | **35N** | **40N** | **45N** | **50N** |
| --- | --- | --- | --- | --- | --- | --- | --- |
| 1 | 15.3 | 22.3 | 25.7 | 32.3 | 36.2 | 39.7 | 48.0 |
| 2 | 20.4 | 25.2 | 30.9 | 32.9 | 43.2 | 47.1 | 48.5 |
| 3 | 13.3 | 22.5 | 30.9 | 37.1 | 42.2 | 45.8 | 50.0 |
| 4 | 22.3 | 27.2 | 33.5 | 42.3 | 47.2 | 52.2 | 55.7 |
| 5 | 18.3 | 27.8 | 31.5 | 37.9 | 49.4 | 51.9 | 55.0 |
| 6 | 17.0 | 25.3 | 31.3 | 37.2 | 42.6 | 49.5 | 54.4 |
| 7 | 25.6 | 25.5 | 32.8 | 37.8 | 42.7 | 46.6 | 49.2 |
| 8 | 14.8 | 20.6 | 27.6 | 32.2 | 41.0 | 43.3 | 46.6 |
| 9 | 17.5 | 22.9 | 28.0 | 33.1 | 38.8 | 42.2 | 45.9 |
| 10 | 21.9 | 27.9 | 34.6 | 38.7 | 40.1 | 44.9 | 46.7 |
| Mean | 18.7 | 24.7 | 30.7 | 36.2 | 42.4 | 46.3 | 50.0 |
| Standard error | 1.2 | 0.8 | 0.9 | 1.1 | 1.2 | 1.3 | 1.2 |

Data showed a normal distribution (Shapiro-wilk test, p<0.05).

Intraclass correlation coefficient: 0.94 (0.85-0.99, p<0.05).

*Repeatability test #2*: 20N load applied 12 times to the 7-sensor insole.

**Output of the 7 sensors for a 20N load.**

| **Loading times** | **Sensor 1** | **Sensor 2** | **Sensor 3** | **Sensor 4** | **Sensor 5** | **Sensor 6** | **Sensor 7** |
| --- | --- | --- | --- | --- | --- | --- | --- |
| 1 | 21.3 | 19.8 | 21.7 | 20.0 | 19.0 | 17.9 | 22.7 |
| 2 | 21.2 | 20.3 | 23.2 | 21.5 | 18.2 | 18.4 | 22.9 |
| 3 | 21.9 | 18.7 | 24.5 | 21.6 | 18.7 | 19.8 | 23.1 |
| 4 | 20.4 | 20.8 | 21.3 | 21.0 | 23.1 | 19.6 | 20.2 |
| 5 | 20.5 | 17.6 | 21.9 | 17.2 | 22.5 | 23.5 | 20.4 |
| 6 | 20.2 | 19.1 | 20.5 | 20.4 | 22.3 | 20.6 | 19.7 |
| 7 | 23.0 | 20.9 | 22.5 | 21.1 | 23.6 | 21.1 | 22.4 |
| 8 | 21.7 | 21.4 | 21.5 | 20.2 | 23.3 | 21.6 | 20.6 |
| 9 | 22.0 | 21.2 | 24.6 | 21.4 | 18.6 | 22.1 | 19.7 |
| 10 | 20.9 | 21.0 | 22.4 | 21.2 | 22.1 | 22.1 | 21.6 |
| 11 | 21.5 | 18.8 | 19.1 | 20.5 | 18.2 | 23.1 | 21.4 |
| 12 | 22.7 | 21.1 | 22.4 | 22.0 | 21.4 | 22.2 | 21.3 |
| Mean | 21.5 | 20.1 | 22.1 | 20.7 | 20.9 | 21.0 | 21.3 |
| Standard error | 0.3 | 0.4 | 0.4 | 0.4 | 0.6 | 0.5 | 0.4 |

Data showed a normal distribution (Shapiro-wilk test, p<0.05)

Intraclass correlation coefficient: 0.56 (-0.14-0.91, p<0.05).
