# Supplementary material for: Random forest algorithms to classify frailty and falling history in seniors using plantar pressure measurement insoles: a large-scale feasibility study
Source: BMC Geriatr. 2022 Sep 12;22:746. doi: 10.1186/s12877-022-03425-5 (PMC9469527; doi:10.1186/s12877-022-03425-5)
Supplement: Supplementary file 3 — Additional file 3. [file 12877_2022_3425_MOESM3_ESM.docx]

**Supplementary Material 3.**

**
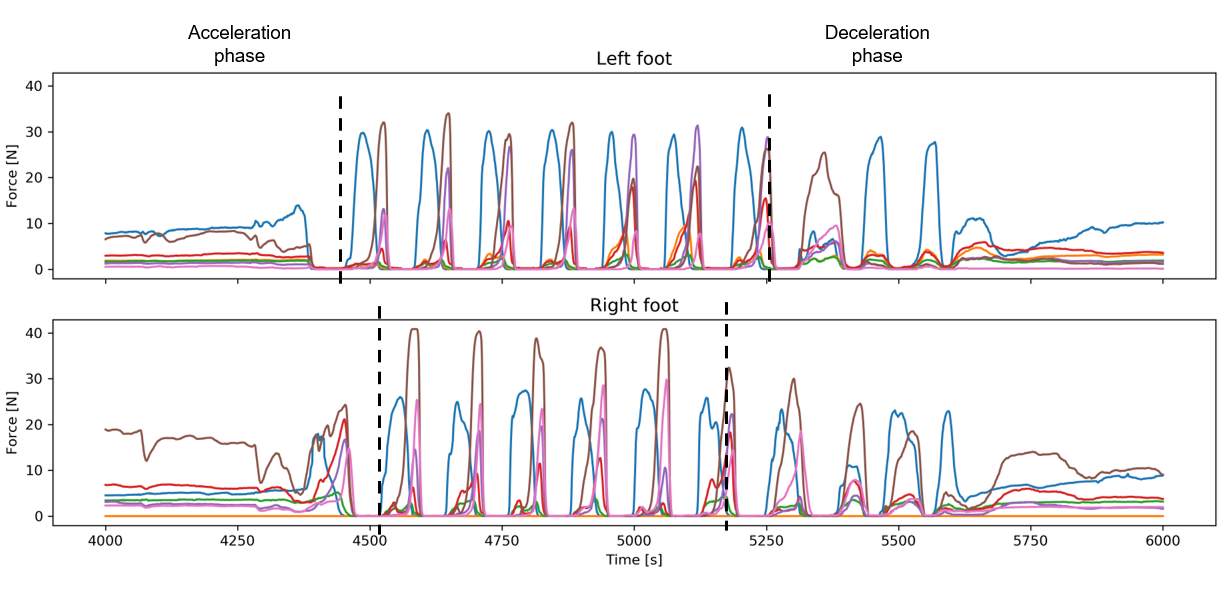
**

**Example of raw plantar pressures collected during the walking test.** Pressures between the dotted lines were kept for isolating steps and extracting data features. Pressures belonging to the acceleration and deceleration phases were not included in the analysis. Blue: heel. Orange: lateral midfoot. Green: center of the midfoot. Red: lateral midfoot. Purple: center of forefoot. Marron: medial forefoot. Pink: big toe. Dotted lines: start and the endpoint of the reduced data.
