# Supplementary material for: Random forest algorithms to classify frailty and falling history in seniors using plantar pressure measurement insoles: a large-scale feasibility study
Source: BMC Geriatr. 2022 Sep 12;22:746. doi: 10.1186/s12877-022-03425-5 (PMC9469527; doi:10.1186/s12877-022-03425-5)
Supplement: Supplementary file 4 — Additional file 4. [file 12877_2022_3425_MOESM4_ESM.docx]

**Supplementary Material 4.**

**Summary of balanced accuracies and weighted F1-scores of random forest models tested with nested-cross validation method.**

|  | Frailty predictions | | Fall predictions | |
| --- | --- | --- | --- | --- |
|  | Accuracy | F1-score | Accuracy | F1-score |
| Whole population  (N = 712) | 0.75±0.04 | 0.77±0.03 | 0.57±0.05 | 0.62±0.03 |
| Age-group: | | | | |
| ≥65 years (N = 585) | 0.76±0.04 | 0.77±0.04 | 0.55±0.05 | 0.60±0.03 |
| 65-69 years (N = 198) | 0.68±0.11 | 0.78±0.05 | 0.60±0.09 | 0.67±0.06 |
| 70-74 years (N = 153) | 0.69±0.09 | 0.71±0.07 | 0.49±0.10 | 0.53±0.08 |
| ≥ 75 years (N = 234) | 0.71±0.06 | 0.71±0.06 | 0.54±0.08 | 0.58±0.07 |
| Sex: | | | | |
| Women (≥ 65 years, N = 409) | 0.72±0.04 | 0.74±0.04 | 0.56±0.06 | 0.61±0.04 |
| Men (≥ 65 years, N = 176) | 0.78±0.07 | 0.79±0.06 | 0.50±0.08 | 0.56±0.06 |
| Frail state: | | | | |
| Frail (N = 203) |  |  | 0.49±0.08 | 0.53±0.07 |
| Non-frail (N = 509) |  |  | 0.58±0.06 | 0.64±0.04 |

Results are presented as mean ± standard deviation.
