# Supplementary material for: Random forest algorithms to classify frailty and falling history in seniors using plantar pressure measurement insoles: a large-scale feasibility study
Source: BMC Geriatr. 2022 Sep 12;22:746. doi: 10.1186/s12877-022-03425-5 (PMC9469527; doi:10.1186/s12877-022-03425-5)
Supplement: Supplementary file 5 — Additional file 5. [file 12877_2022_3425_MOESM5_ESM.docx]

**Supplementary Material 5.**


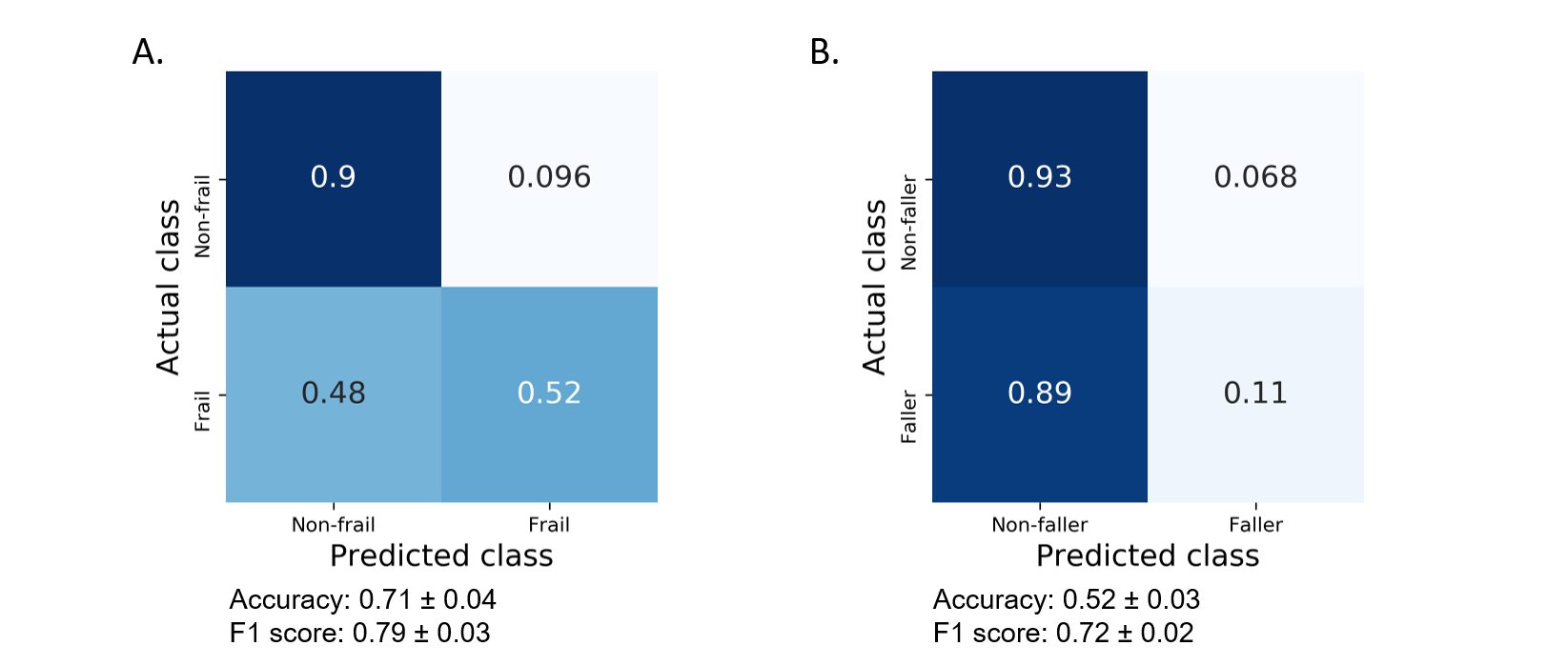


**Linear regression classifier performance of the frailty state and falling history for the whole population presented as confusion matrices.** A: frailty state. B: falling history.


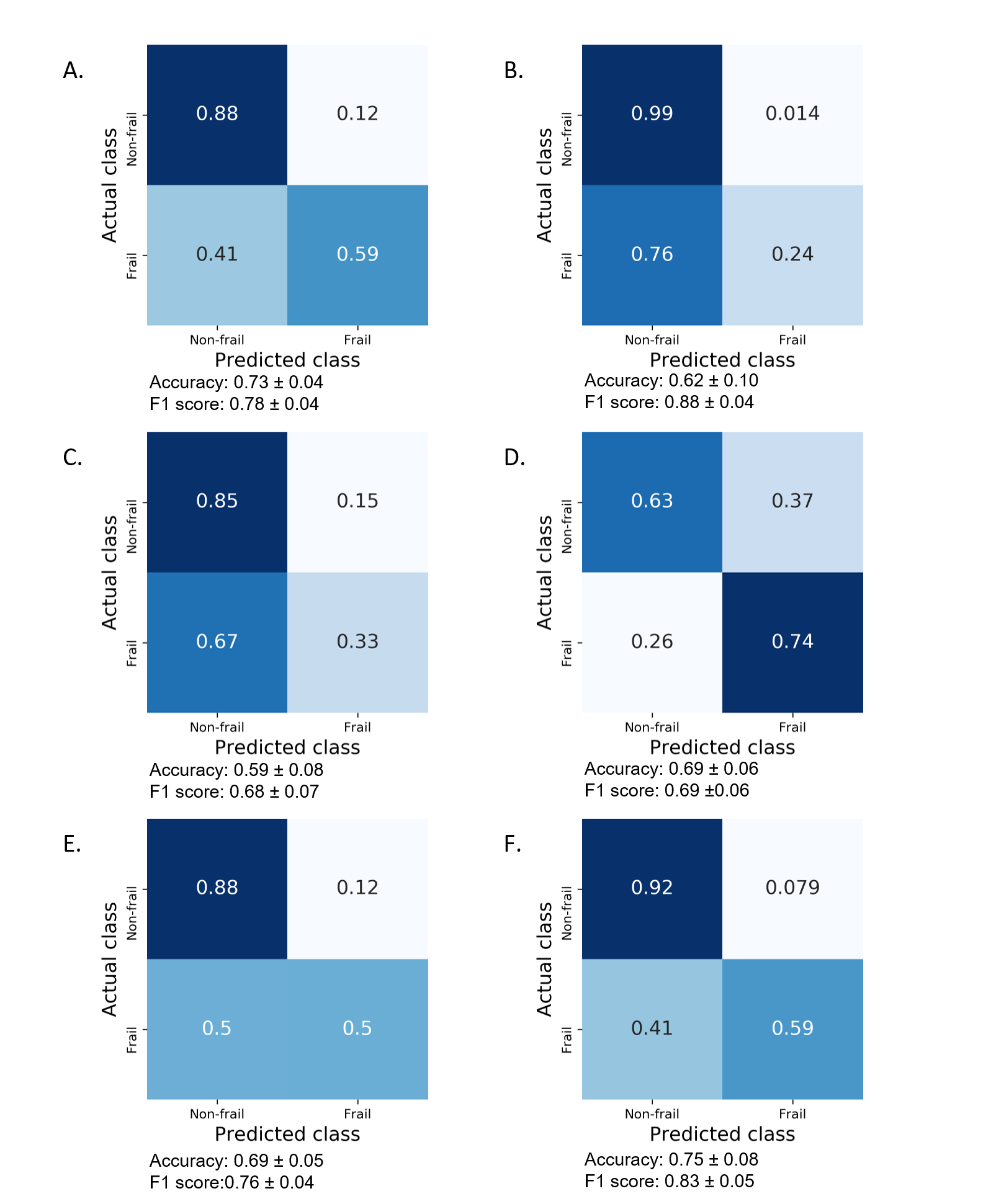


**Linear regression classifier performance of the frailty state classifiers presented as confusion matrices for all the subgroups.** A: aged ≥65 years old. B: aged between 60–69 years old. C: aged between 70–74 years old. D: aged ≥75 years old. E: women only (≥ 65 years). F: men only (≥ 65 years).

**Important features for linear regression classifier in classifying frailty in the whole population and relative to each subgroup.**

The table continues on next page.

The table continues on next page.

For the whole population models, features are listed from top to bottom in the same order as they ranked (see the top 10 rows). Then, the new important features found in the 1) subjects aged ≥65 years old, 2) subjects aged between 60–69 years old, 3) subjects aged between 70–74 years old, 4) subjects aged ≥75 years old, 5) women aged≥ 65 years, and 6) men aged ≥65 years analyses appear in the 31 remaining rows.

**Random forests vs. Logistic regressions**

Compared to the random forest models, logistic regressions systematically show lower accuracy scores.

Logistic regression models also result in a higher number of “important features” (41 vs. 27 for random forests). This observation taken together with the lower accuracy scores also noted for the logistic regression models may reflect a greater difficulty of the logistic models in converging towards the most important variables. Fifteen features were identified as essential features in more than one analysis (12 random forests). Features from the category “peak analysis and area under the curves” accounted for 12 of the 41 identified important features and 2 of the top-10 essential features (random forest: 10/27 and 3/10, respectively). Features extracted from the wavelet analysis accounted for 11 of the 41 identified important features and 6 of the top-10 essential features (random forest: 10/27 and 6/10, respectively). Features from the category “1-foot COP trajectory analysis” accounted for 8 of the 41 identified important features and 2 of the top-10 essential features (random forest: 4/27 and 0/10, respectively). Contrasting with the observations done for the random forest models, none of the features was identified as essential in all logistic regression analyses (i.e., whole population analysis and all subgroup analyses).
