# Supplementary material for: Random forest algorithms to classify frailty and falling history in seniors using plantar pressure measurement insoles: a large-scale feasibility study
Source: BMC Geriatr. 2022 Sep 12;22:746. doi: 10.1186/s12877-022-03425-5 (PMC9469527; doi:10.1186/s12877-022-03425-5)
Supplement: Supplementary file 6 — Additional file 6. [file 12877_2022_3425_MOESM6_ESM.zip › 6-Supplementary Material 6/Data-Code/Legends.docx]

**Supplementary Material 6:**

Dataset: Data.csv

Code: Analysis.py.
